# The Persistence of the Self over Time in Mild Cognitive Impairment and Alzheimer's Disease

**DOI:** 10.3389/fpsyg.2018.00094

**Published:** 2018-02-20

**Authors:** Lynette J. Tippett, Sally C. Prebble, Donna Rose Addis

**Affiliations:** ^1^School of Psychology, The University of Auckland, Auckland, New Zealand; ^2^Centre for Brain Research, The University of Auckland, Auckland, New Zealand; ^3^Brain Research New Zealand, Auckland, New Zealand

**Keywords:** self-persistence, diachronic unity, Alzheimer's disease, amnestic mild cognitive impairment, narrative identity, phenomenological continuity, semantic continuity

## Abstract

Diachronic unity is the belief that, despite changes, we are the same person across the lifespan. We propose that diachronic unity is supported by the experience of remembering the self over time during episodic recall (i.e., phenomenological continuity). However, we also predict that diachronic unity is also possible when episodic memory is impaired, as long as the ability to construct life narratives from semantic memory (i.e., semantic continuity) is intact. To examine this prediction, we investigated diachronic unity in Alzheimer's Disease (AD) and amnestic mild cognitive impairment (aMCI), two conditions characterised by disrupted phenomenological continuity. If semantic continuity is also altered in these conditions, there should be an associated deterioration in diachronic unity. Participants with AD, aMCI, and healthy controls (HC) completed a self-persistence interview measuring diachronic unity (beliefs about self-persistence, explanations for stability/change). Semantic continuity was assessed with a life-story interview measuring autobiographical reasoning (self-event connections), and coherence (temporal/thematic/causal) of narratives. Our results highlight a complex relationship between semantic continuity and diachronic unity and revealed a divergence between two aspects of diachronic unity: AD/aMCI groups did not differ from HC in continuity beliefs, but AD explanations for self-persistence were less sophisticated. Semantic continuity was most impaired in AD: their narratives had fewer self-event connections (vs. HCs) and lower temporal/thematic coherence (vs. HC/aMCI), while both AD/aMCI groups had lower causal coherence. Paradoxically AD participants who scored higher on measures of beliefs in the persistence of the core self, provided less sophisticated explanations for their self-persistence and were less able to explore persistence in their life narratives. These findings support the importance of semantic continuity to diachronic unity, but suggest a more nuanced and multifaceted relationship than originally proposed in our model. In AD, diminished life narratives that retain features of cultural life scripts are sufficient for strong subjective beliefs of self-persistence, but not for sophisticated explanations about persistence. Better semantic continuity, with the ability to weave high-quality life narratives, may scaffold the capacity to understand and explain one's diachronic unity, but this produces less surety about self-persistence.

## Introduction

**Interviewer:** Do you feel that you are the same person now as you were when you were in your early 20s?

***Participant:** Yes, oh yes! I don't know, I don't know why. I feel like I'm the same*.

**Interviewer:** What do you think makes you the same person? How would you explain how one and the same person could act in so many different ways but still be the same person?

***Participant:** I feel the same*. - Extract from self persistence interview (Participant 010b from AD group, MMSE = 10).

One of the most intriguing aspects of the self is its persistence and unification across time. Known as *diachronic unity* (see Appendix A for a glossary of italicised terms), this does not imply that one is unchanged, but rather expresses a deep conviction that, despite change, one continues to be the same person now as in the past, and will continue to be the same person into the future (Sani, 2008; Klein, 2010; Prebble et al., 2013). Intuitively, *autobiographical memory* (AM) is critical to this experience of diachronic unity. The act of remembering oneself in the past instantly links the present individual to their past self: mentally, emotionally, and experientially. Just as I can know that my current experiences are my own because my subjective experience tags them as such, so too I can know that my episodically remembered past was experienced by “me.” This sentiment is clearly articulated in Locke (1964/1970) claim about the relationship between consciousness and identity: “as far as this consciousness can be extended backwards to any past action or thought, so far reaches the identity of that person; it is the same self now it was then; and it is by the same self with this present one that now reflects on it, that that action was done.” (p. 181).

Indeed the link between AM and sense of self appears so clear that it is difficult to imagine a theory of diachronic unity that does not incorporate a mnemonic element. Moreover, it has been argued that the experience of continuity is a critical feature of diachronic unity, distinguishing it from synchronic unity (i.e., the unification of self in the present moment) that is supported by short-term memory (Tye, 2003; Rashbrook, 2013). The few studies that have explored people's subjective beliefs about their diachronic unity suggest that a firm sense of persistence across time is fairly ubiquitous both across cultures and across the lifespan (e.g., Troll and Skaff, 1997; Chandler et al.'s, 2003); they also point to the potentially catastrophic consequences of any deterioration in these beliefs (Chandler et al.'s, 2003; Hsiao et al., 2013). To date, however, there has been little empirical research into the cognitive mechanisms that serve to support diachronic unity, and what role is played by AM. In this paper we report the first systematic exploration of diachronic unity in two groups of individuals with impaired episodic memory: those with amnestic mild cognitive impairment (aMCI) and probable Alzheimer's Disease (AD).

**Figure 1:**
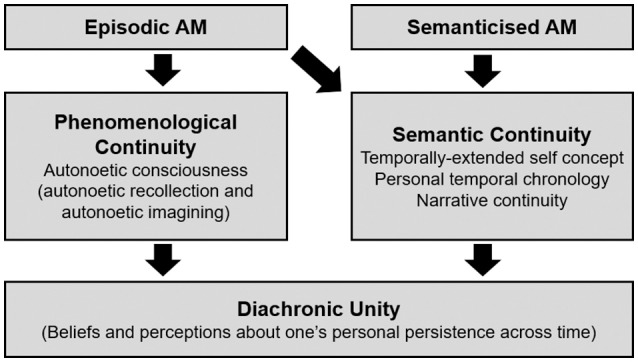
Model of the temporally extended self, showing two autobiographical memory mechanisms for continuity, and their proposed relationship to diachronic unity.

We recently proposed two parallel mechanisms through which AM may serve to support diachronic unity (Prebble et al., 2013; see Figure 1). First, remembering one's past experiences—a form of AM known as *episodic memory*—is associated with projecting oneself back into the past to consciously re-experience discrete moments in time (Wheeler et al., 1997; Tulving, 2002; Vandekerckhove and Panksepp, 2009). Known as *autonoetic consciousness*, we have argued this aspect of episodic memory affords a sense of *phenomenological continuity* (Addis and Tippett, 2008; Prebble et al., 2013). A number of single case reports in the clinical literature of densely amnesic individuals support this view (Tulving, 1985; Wilson and Wearing, 1995; Postle, 2009). Patient C.W. provides a striking example; he lacks all episodic memories and constantly reports that he is conscious for the first time: “…this is the first sight I've had, the first taste I've had (sipping his coffee) it's like being dead” (Wilson et al., 2008). Patient C.W. and other cases clearly demonstrate a severe discontinuity of phenomenological experience. Yet there have been few systematic explorations of how these deficits might affect these individuals' subjective beliefs about their diachronic unity (Troll and Skaff, 1997), including their ability to logically account for how and why they remain the same person across time (Chandler et al.'s, 2003).

The loss of episodic memory and autonoetic consciousness disrupts phenomenological continuity which in turn impacts subjective beliefs of diachronic unity. However, it is possible that even in the context of disrupted phenomenological continuity, diachronic unity can be supported via a second mechanism: *semantic continuity*. Rather than being experiential, this form of continuity is knowledge-based, mediated by *semantic memory*, an abstract and conceptual type of AM that synthesises large amounts of information (including entire life periods and repeated events) (Conway and Pleydell-Pearce, 2000). Critically, semantic memory enables one to create *narratives*—life stories that explain “who I was and how I came to be who I am” and includes *temporally-extended self-concept* and *personal temporal chronology*—from which semantic continuity emerges (Klein et al., 2003; Piolino et al., 2006; Rathbone et al., 2009). Interestingly the neuropsychological case studies of dense episodic amnesia not only illustrate a severe discontinuity of phenomenological experience, but hint at a form of diachronic unity—a retained belief about their own *self-persistence* (Medved and Brockmeier, 2008; Postle, 2009; Philippi et al., 2012). While clearly distressed that they are unable to remember their past, they express an appreciation that they *ought* to possess a mnemonic link to the past, which appears to presuppose some notion of themselves as temporally-extended beings. Further, although comments suggest a feeling of being marooned in the present moment (Postle, 2009), their speech and thoughts are not restricted to the present moment as one might expect if one lacked any sense of temporal extension. H.M. not only repeatedly retold the few (semanticised) AMs he had retained (Corkin, 2002), he also expressed emotions that related to his past and his future; anxiety about what might have happened before but could not recall, and concern about how his present actions might impact upon the future. Their words and actions hint at an emotional and behavioural connection to their past and future in the absence of any episodic memories that could provide these links (for a similar model, see Bluck and Liao, 2013). This suggests that although the ability to mentally project oneself into the past may contribute to diachronic unity, it may not be essential.

To examine these proposed mechanisms through which AM may support diachronic unity in the following study we conducted a formal investigation of diachronic unity in two groups with impaired episodic memory and autonoetic consciousness: individuals with aMCI and those with probable early-moderate AD (Rauchs et al., 2007; Addis et al., 2009; Ally et al., 2009; Hudon et al., 2009; Irish et al., 2010). If autonoetic recollection of event memories is essential for diachronic unity, the marked deterioration in episodic memory and autonoetic consciousness in those with AD should lead to a corresponding deterioration in their subjective beliefs about their self-persistence, and to a lesser extent in those with aMCI where impairments of autonetic consciousness are less severe (Hudon et al., 2009). Alternatively, if beliefs about self-persistence have not weakened in these groups, this would provide some evidence that episodic memory, and the phenomenological continuity it affords, is not the sole mechanism supporting diachronic unity. To this end, we examined how aspects of semantic continuity, specifically *narrative identity* and personal temporal chronology, is affected in individuals with aMCI and AD, and how any such deterioration in turn impacts upon diachronic unity. These two groups provide an excellent opportunity to investigate these questions. As noted above, there are consistent findings indicating both deficits in episodic memory and autonoetic consciousness in aMCI and early AD, as well as preserved semantic AM in aMCI (Murphy et al., 2008). In contrast there is divergence in the literature as to whether semantic AM is relatively preserved in early-AD (Gilboa et al., 2005; Martinelli et al., 2013), or showing some evidence of decline (e.g., Addis and Tippett, 2004).

Two main studies have explored diachronic unity, using different approaches to assess subjective beliefs about one's persistence across time. Troll and Skaff (1997) examined beliefs about self-persistence in older adults by asking two simple questions: “In what ways have you always been the same?” and “In what ways have you changed over the years?” They attempted to separate subjective beliefs about whether one's “core self” or essential person was still the same (e.g., “I feel that my essence has always been the same”) from perceived changes in the content of self-concept (e.g., characteristics and traits) over time. Beliefs about one's essential person directly captures phenomenological sense of self-persistence, while beliefs about content of self-captures persistence of the self-concept (i.e., temporally-extended self-concept which likely impacts one's beliefs about their diachronic unity.

Chandler et al.'s (2003), in contrast, asked the young people in their study to articulate an explanation for their self-persistence, following an exercise with a fictional character. Participants were first presented with a literary passage involving a character who undergoes considerable personal change (e.g., Jean Valjean in *Les Miserables*), and asked to explain why the character was still the same person by the end of the story. Participants were then asked to reflect on the ways that they had changed throughout their own lives, and explain why they were fundamentally the same person despite these changes.

It is not clear how these two ways of approaching beliefs about diachronic unity might relate. For example, is a logical rationale for one's self persistence necessary in order to sustain a firm belief about one's persistence across time? There is evidence from Chandler et al.'s (2003) work, that in young people, lack of a clear explanation for self-persistence may be associated with suicidal tendencies, suggesting that these aspects of diachronic unity may be linked.

In this study, we combined both approaches to create a comprehensive *self-persistence interview* that assesses diachronic unity. The general aim of the interview was to guide participants to think about, and describe, their subjective beliefs about how they have changed and remained the same over their lives, whether they continue to consider themselves to be the same person, and the reasons underlying these beliefs. Instead of using a fictional story to elicit thoughts about stability and change, we primed participants by having them complete two versions of the Twenty Statements Test (TST; Kuhn and McPartland, 1954), one of which required participants to describe who they are in the present using 20 “I am…” statements, and the other of which asked them to describe who they were in their early 20s. (The results of the two TSTs are not discussed in this paper.) The aim of this method was to provide a concrete point of comparison for the older participants involved in the present study, inviting a direct comparison between their own descriptions of themselves in the present and past, before proceeding to the self-persistence interview. This provided a more direct way to engage our participants, particularly those with AD, in a discussion about diachronic unity.

A proposed contributor to diachronic unity, semantic continuity is characterised by the ability to construct a coherent, meaningful life story out of one's memories that highlights personal temporal chronology. A central assumption of narrative identity theory is that this narrative process is an adaptive ability, possibly through its link to creating sense of continuity (Habermas and Bluck's, 2000; McAdams, 2001). More recent investigations, however, have challenged the uniform benefit of narrative identity, suggesting instead that the benefit of narrative processes like *autobiographical reasoning* may depend on many complex factors (McLean and Mansfield, 2011; Greenhoot and McLean, 2013; Habermas and Köber, 2014). Nevertheless, if narrative processes are linked to sense of persistence, this suggests that those who are able to construct “better” life narratives should also possess a stronger sense of diachronic unity. To date very little research has examined how narrative identity and autobiographical reasoning may relate to diachronic unity. Habermas and Köber (2015) found that the amount of autobiographical reasoning in the life narratives of healthy adults correlated positively with a sense of self persistence (measured using a four item-scale assessing feeling of familiarity with themselves in the past), but only in those who had experienced serious disruptions in their lives (“biographical disruptions”) in the previous 4 years. The authors suggest that narrative identity may be important to self-persistence only when this sense of continuity is challenged in some way (see also Habermas and Köber, 2014).

No studies have yet investigated whether a loss of AM may constitute such a challenge, or whether constructing a life story capable of supporting diachronic unity is even possible in the face of such memory loss. The few studies that have attempted to examine narrative identity in AD suggest that limited narrative abilities may remain even in the face of severe AM deficits (Mills, 1997; Usita et al., 1998; Surr, 2006). Although these studies did not provide any formal analysis of the use of autobiographical reasoning or the global coherence of narratives, their reports provide some hints about how the quality of the narratives may be affected. Surr notes that the majority of their AD participants narrated stories which “integrated the whole of their life from the past to the present…” and appeared to be “…setting their present experiences in the context of their past in order to maintain a sense of self” (Surr, 2006, p. 1727–1728). This description is strikingly similar to descriptions of autobiographical reasoning, providing a tentative suggestion that this capacity may also be preserved to some extent in early stages of the disease.

These studies also touch on some difficulties with the narratives created by those with AD, including an inability to narrate all or part of a life story (Surr, 2006), producing stories that were fragmented and repetitive (Mills, 1997), or that lacked detail in the description of specific events (Usita et al., 1998). Usita et al. found that although the life stories of healthy participants were consistently chronologically ordered and included culturally important life events (e.g., marriage, having children), the stories of those with AD were not. Using Habermas and Bluck's (2000) terminology, these reports seem to suggest that the life stories of those with AD lack temporal coherence and the framework for a cultural concept of biography (i.e., a cultural life script). Another study, however, found that participants with dementia were more likely to include culturally important life events in their stories than healthy adults (Fromholt and Larsen, 1991), suggesting that this important organisational structure for AM may be fairly resilient in AD. As neither of these studies provided any measures of temporal coherence or the cultural life script, it is difficult to assess these conflicting claims.

A goal for the present study was to provide formal measures of semantic continuity by assessing the extent to which narrative processes—autobiographical reasoning and global coherence including personal temporal chronology—are affected in aMCI and AD. More importantly, we aimed to address the relationship of these measures of semantic continuity with diachronic unity. If the quality of the life story, and/or the ability to construct a personal temporal chronology, are indeed important for diachronic unity, those who score higher on measures of global coherence and autobiographical reasoning would also be expected to express more certainty about their persistence across time.

To assess the use of autobiographical reasoning in life stories, we examined self-event connections. Self-event connections involve the creation of connections between one's conceptual self and the events of one's life (Pasupathi and Mansour, 2006; McLean, 2008; McLean and Fournier, 2008). They are suggested to create continuity either by emphasising elements of personal stability over time (stability connections), or by explaining how and why an individual has changed as a result of life events (change connections) (Pasupathi and Mansour, 2006; Pasupathi et al., 2007; McLean, 2008). Autobiographical reasoning was predicted to be particularly crucial for individuals with AD who face significant challenges, or disruptions, with their diagnosis.

We also examined the global coherence of life stories (Habermas and Bluck's, 2000; Habermas, 2011) as a second marker of the quality of semantic continuity. Whereas, autobiographical reasoning involves links made between the self and particular events within the story, global coherence relates to the ability to link and integrate many events into a cohesive whole. Habermas and Bluck's (2000) describe four main elements that contribute to the global coherence of a life narrative. Causal coherence involves explaining how different elements of the story led to, brought about or were dependent upon other elements, including how aspects of the self were affected by events in one's life, and how one's personality contributed to the events that occurred. Thematic coherence is the extent to which a narrative provides interpretive links within the story by identifying higher-level similarities, motifs, and metaphors that provide meaning and cohesion to the story. In addition, global coherence includes two features that facilitate the personal temporal chronology of the narrative. Temporal coherence probes the integrity of personal temporal chronology, specifically the ability to temporally locate, order, and sequence events in relation to each other and within the wider context of one's life. Cultural concept of biography concerns the degree to which one's story incorporates culturally-accepted rules about which elements are important to include in one's life story. These cultural expectations around the life story are suggested to form a cultural life script, which provides the skeletal structure for life stories.

In the present study, we aimed to provide the first systematic exploration of sense of diachronic unity in those with aMCI and mild-to-moderate probable AD. By using a range of novel and pre-existing measures of diachronic unity, we sought to examine how these different ways of conceptualising self-persistence might relate, and how they may be differentially affected by memory deterioration in these conditions. More specifically, our model (Prebble et al., 2013) proposes two mechanisms through which AM may contribute to individuals' subjective beliefs about persistence: phenomenological continuity and semantic continuity. Based on this model, we predicted that although AD (and to a lesser extent aMCI) is characterised by a deterioration in phenomenological continuity associated with episodic memory deficits, this alone should not lead to deterioration in diachronic unity, provided that semantic continuity is well-preserved. The evidence reviewed above, however, indicates that aspects of semantic continuity in AD (particularly narrative processes such as autobiographical reasoning and global coherence including personal temporal chronology) may nevertheless be altered, which should in turn lead to deterioration in diachronic unity.

## Methods

### Participants

#### Patient groups

The AD and aMCI groups each comprised 15 individuals. Inclusion criteria included formal medical diagnosis of either probable AD or aMCI (Albert et al., 2011; McKhann et al., 2011), fluency in English, no acquired language difficulties preventing communication, and being capable of giving informed consent. Potential participants were excluded if they had a history of major head injury, cerebrovascular disease, neurological abnormality (other than AD/aMCI), alcoholism or drug dependence, psychiatric illness, or prolonged use of psychiatric medication. Participants in the AD group were required to have a dementia severity of mild-to-moderate, with a score of 10 or above (out of 30) on the Mini-Mental State Examination (MMSE; Folstein et al., 1975). Individuals meeting these criteria were identified by clinicians during routine memory clinic appointments (at outpatient memory clinics run through two public hospitals and two private memory clinics in Auckland, New Zealand), and those who expressed an interest in the study were contacted directly by the researcher.

#### Control group

The healthy control (HC) group consisted of 25 older adults recruited through advertisements distributed through retirement villages and community groups involving older people. The same exclusion criteria described above also applied to the HC group. In order to screen for undiagnosed dementia, HC participants in the control group were required to have an MMSE score of 25 or greater (Folstein et al., 1975). In addition, any potential participants who described difficulties in memory, appeared forgetful in the interview, or performed poorly on the MMSE memory component, were screened for memory impairment with the Rey Auditory Verbal Learning test (Rey, 1964). Only one participant was excluded on this basis, with performance below age-stratified norms (Strauss et al., 2006).

The demographic characteristics of the three groups are summarised in Table 1. The groups did not differ significantly in terms of sex, $\chi_{\left(2\right)}^{2}$ = 0.69, *p* = 0.71, age, *F*_(2, 52)_ = 1.82, *p* = 0.17, or years of education, *F*_(2, 52)_ = 2.39, *p* = 0.10. As expected, there was a significant group difference on general cognitive performance (MMSE score), *F*_(2, 18.11)_ = 14.65, *p* < 0.001 (Brown-Forsythe *F* test for groups with unequal variance), and verbal fluency (Controlled Oral Word Association score), *F*_(2, 52)_ = 6.17, *p* = 0.004. *Post-hoc* Games-Howell tests confirmed that MMSE scores of both memory-impaired groups (AD: *p* < 0.001; aMCI: *p* = 0.046) were significantly lower than the HC group, and the AD group was significantly lower than the aMCI group (*p* = 0.002). The AD group also scored significantly lower than both the HC and aMCI groups (*p* ≤ 0.01) on COWA, although the aMCI group did not differ significantly from the HC group (*p* = 1.0).

**Table 1:** Demographic characteristics of the study participants.

	**HC group**	**aMCI group**	**AD group**
Number	25	15	15
Sex (F/M)	13/12	6/9	8/7
	M (SE)	M (SE)	M (SE)
Age (years)	82.15 (1.51)	78.09 (1.61)	77.7 (2.79)
Range	67–98	64–87	58–95
Education (years)	14.58 (0.54)	13.97 (0.81)	12.6 (0.65)
Range	9–19.5	10–19	7–18.5
MMSE	28.2 (0.26)	26.87 (0.46)^*^	20.87 (1.42)^*^
Range	26–30	24–30	10–27
COWA	37.28 (2.43)	37.07 (2.58)	25.6 (2.46)^#^
Range	20–67	19–51	14–44

*M, Mean; SE, Standard Error of the Mean; MMSE, Mini-Mental State Examination; COWA, Controlled Oral Word Association test; HC, Healthy control; aMCI, amnestic Mild Cognitive Impairment; AD, Alzheimer's disease*.

* *Significantly lower than HC group*.

# *Significantly lower than HC and aMCI groups*.

### Measures

#### Self-persistence interview

This interview was designed to assess diachronic unity, and incorporated elements from the approaches used by Chandler et al.'s (2003) and Troll and Skaff (1997); note that we have renamed the measures (as indicated by footnotes) to maintain the consistency of the terminology used within the present paper. Responses were transcribed verbatim and the entire self-persistence interview transcripts were analysed when applying each of the separate coding elements described below.

##### Perceived persistence^1^ (Troll and Skaff, 1997)

Participants were asked whether they believed that they were still the same person as they were in their early 20s. The yes/no responses to this question were recorded; however some participants responded “yes and no” or “maybe.” “Yes” responses were coded “3,” “yes and no” and “maybe” responses coded “2,” and “no” responses coded “1.” For analyses involving a dichotomous variable, mid-way answers were collapsed with the “no” responses. If participants provided an explanation for their answer, this text was transcribed verbatim, and coded as part of the other sections of the interview, as appropriate (see below).

##### Persistence of the I-self and Me-self^2^ (Troll and Skaff, 1997)

Participants were next asked how they believed they had changed, and how they believed they had remained the same since they were in their early 20s. An independent coder read through the responses to identify elements that related to the *I-self* (the essential, core self, the underlying essence of who you are, the inner entity; related to beliefs about phenomenological continuity) vs. the *me-self* (the attributes one uses to describe oneself, e.g., traits, attributes, physical and personality descriptions, behaviour, likes and dislikes, values and beliefs; related to beliefs about semantic continuity). The coder then scored each set of responses using two three-point scales (an I-self and a me-self scale) based on a coding method from Troll and Skaff. This method assessed perceptions of change vs. persistence, with higher scores indicating a greater perception of persistence: a score of “1” indicated a perception of fundamental change, “2” indicated some change, and “3” indicated a firm perception of persistence (no change) (see Table 2).

**Table 2:** Summary of the coding scheme for the I-self and me-self questions of the self-persistence interview.

**Score**	**I-self persistence subscale**	**Me-self persistence subscale**
1. Fundamental change	Indicates a perception of fundamental change in the core self (e.g., “The person I used to be is no longer there”)	Indicates a perception of fundamental change in one's attributes (e.g., “I have changed in so many since I was young – personality, the way I look and act, the people I associate with”)
2. Some change	Indicates a perception of some change in the core self or uncertainty about core continuity (e.g., “Not exactly the same person…You're different but the same—it's hard to explain”)	Indicates a perception of some change in attributes (e.g., “In my intellectual interests I'm pretty much the same; but I've changed in appearance.”)
3. No change	Indicates a firm perception of continuity in the core self (e.g., “I feel my essence has always been the same.”)	Indicates a firm perception of stability in attributes. (e.g., “I've always been calm, composed…independent. I'm just the same now.”)

*Coding scheme adapted from Troll and Skaff (1997)*.

##### Persistence explanation^3^ (Adapted from Chandler et al.'s, 2003)

The last part of the self persistence interview asked participants to explain the reasons why, given the many changes that had taken place in their lives, they still considered themselves to be the same person:

What I now want you to do now is to think about the reasons that you consider yourself to be the same person that you were when you were a young adult. What do you think makes you the same person? I want you to explain these reasons. How would you explain how one and the same person could act in so many different ways but still be the same person?

Responses were coded using a simplified version of Chandler et al. coding scheme that used a five-point scale to grade the complexity or sophistication of the response. Very simple responses (level 1) highlight easily identifiable, surface qualities of the self that have remained the same across time (e.g., particular physical features or simple personality traits) while ignoring or downplaying elements of the self that have changed. More sophisticated answers (which scored higher) are those which integrate ways that the self may have changed while also identifying factors that maintain stability. Persistence explanations scored at the highest levels of sophistication identify a core element of the self which has remained unchanged despite (perhaps considerable) change in more surface attributes (level 4), or demonstrate a meta-awareness of the paradox of persistence by weighing up a number of possible explanations that could account for change and persistence in the self (level 5) (see Table 3).

**Table 3:** Summary of coding scheme used to score on the persistence explanation question of the self-persistence interview.

**Level of sophistication**	**Description**	**Examples**
Level 1	Response describes simple, surface elements of the self that have not changed, while ignoring aspects that have changed.	I just seem to do the same things. If people talk to me I talk to them, if people need help I help them. Always been the same.
Level 2	Response attempts to engage with the problem that people appear to change by suggesting that apparent changes are in fact aspects of the self that were present from the start but have never been seen until now.	When I was with my ex-husband, it really brought out my angry side. It's still there but now I don't have to be that way so much.
Level 3	Response acknowledges the effects of time as the agent of growth and development of pre-existing traits.	I am the same person, I have just grown. The experiences of my life have brought out a mature version of me.
Level 4	Response attempts to solve the problem of change/continuity by proposing a core, underlying essence of the self which remains unchanged despite change in surface attributes.	Spiritually—your body changes but spiritually you stay the same.
Level 5	Response provides a “meta answer” which indicates that whatever explanation the person may have to the problem of self-continuity is simply one theory among many.	There are many ways you could answer that. I have the same brain. Also, a lot of the attitudes you had when you were little….You remember what you were. This also gives you continuity…Your genes don't change….attitudes stay with you / born in you?

*Coding scheme adapted and simplified from Chandler et al.'s (2003)*.

#### Life story interview (adapted Negele and Habermas, 2010)

This interview elicited a life story which was then assessed for various aspects of semantic continuity. Participants were provided with a very broad initial question, and were then left to tell their story, uninterrupted and in their own words:

*The main thing I would like to do today is for you to tell me your life story. I would like you to tell me about your whole life, from the time you were born until the present time. You might like to tell me about the most important events in your life and the biggest changes. You can tell me things that someone who doesn't know you might like to know about your life*.

This method (originally used with children) was adapted for use with older adults and AD participants by dividing the story into four chapters: childhood (0–14 years), teenage years and early adulthood (15–25 years), middle adulthood (26–50 years), and late adulthood (51-present). These time periods were selected as a compromise between the need for roughly equal life periods, the need for as few time periods as possible to reduce task demands, and the need to cleanly isolate theoretically important memory periods (e.g., the reminiscence bump). Participants were given 10 min to narrate each chapter. A brief summary of the task instructions along with the relevant life period was placed in front of them while they spoke (e.g., “Please tell me about your childhood: Birth to 14 years old”). If they stopped speaking for longer than 15–20 s, prompts were used to remind them of the task and to elicit more information (e.g., “Is there anything else that you can tell me about that period of your life?”) Initial instructions were repeated when necessary. The life stories were coded for a number of markers of narrative identity.

##### Self-event connections

These were defined as any statement which explicitly connected the narrator's self-concept and the events they are narrating (based on definitions developed by Pasupathi and Mansour (2006) and Pasupathi et al. (2007). Two types of self-event connections were identified. Self-event stability connections suggested a pre-existing quality of the self-explained, or was illustrated by, an event (e.g., “I decided to go overseas because I've always been really adventurous”; “This event shows how persuasive I am”). Self-event change connections explained how and why the narrator had changed as a result of life events (e.g., “After that, I became a lot more wary of other people”). The dependent variable was the number of each type of statement used in the life stories.

##### Global coherence

This coding protocol was adapted from an original manual developed by Habermas and Diel (2005; see also Habermas and de Silveira, 2008), translated from German and amended by Reese and Suggate (E. Reese, personal communication, May 31, 2010). The coding scheme required a rater to read through the life story, and assess how well it achieved three aspects of global coherence. The temporal coherence score assessed the degree to which the rater could follow when, and in what order, events within the story took place. The causal coherence score assessed the degree to which the rater understood how the narrator had changed throughout their story (in terms of personality, circumstances, or outlook), and how the events in the story explain this change. The thematic coherence score assessed how well particular elements within the story were linked or positioned in relation to one another. An independent coder rated each life story chapter for each type of coherence, and scored the text on a four-point scale, from 0 (very low coherence) to 3 (very high coherence). Chapter scores were summed to provide a measure of each type of coherence for the life story as a whole.

##### Cultural life script

Life stories were assessed to determine whether they included cultural life script events. This list of events comprised events identified in at least three studies that had surveyed large numbers of individuals to assess which life events were judged to be important to the life stories of a typical individual (Berntsen and Rubin, 2004; Habermas, 2007; Bohn and Berntsen, 2008; Thomsen and Bernsten, 2008; Rubin et al., 2009; Janssen and Rubin, 2011; Tekcan et al., 2012). A total of 29 event categories met this criterion. Some event categories were slightly tailored for a New Zealand context (e.g., “enter day-care” was amended to “enter day-care, kindergarten or preschool”; “College” was changed to “University or Technical Institute”).

Each life story was read by an independent coder to assess whether it contained any mention of each of the event categories on the list (present/absent). It was scored as “present” only if it was a personal event that occurred to the narrator. The total number of event categories mentioned in the life story was tallied, providing a score for the number of cultural life script categories sampled by each participant.

The number of new occurrences of each event category was also counted. Events were counted as new occurrences if they described a new element in the story from within the same life script category (e.g., attending two separate high schools, the birth of successive children). The number of new occurrences was tallied across event categories, providing a score for the number of cultural life script events mentioned by each participant.

Seven events were mentioned so infrequently in the stories that reliability could not be calculated and were therefore removed from the analysis. For each of the remaining 22 event categories, inter-rater agreement for the present/absent ratings across participants was calculated using Cohen's Kappa (see Coding Procedure, below): 4 showed moderate agreement (range:.43 −0.59), 7 showed substantial agreement (range:.64–0.77) and 10 had a near-perfect agreement (range:.82–1.0). The kappa for the most frequently used event (Get a Job/Settle on career) could not be calculated because this event was present in almost every life story, and one coder had not used the “not present” code for any transcripts. Percent agreement between coders was very good (94%), as was the intraclass correlation for the number of occurrences of this event (ICC = 0.96).

#### Awareness of memory deficits

A brief, structured interview was administered to the aMCI and AD groups to assess awareness of memory deficits, based on methods used by Loebel et al. (1990) and Sevush and Leve (1993). The interview consisted of a few simple questions asking whether participants thought they had problems with memory, and whether they suffered from an illness impacting on their memory. Due to time constraints, this brief assessment was favoured over lengthier approaches (see Clare et al., 2005; Souchay, 2007).

Responses were scored on a three-point scale using a scoring method adapted from Sevush and Leve (1993): responses were scored “2” if they suggested a high level of awareness of deficits (i.e., they acknowledged problems with memory and an awareness of its severity); “1” if they indicated some awareness of problems but underestimated the severity (e.g., they acknowledged minor problems, but suggested these were akin to others their age or had always been a problem); and “0” if they showed a complete lack of awareness of memory impairments (e.g., denied any problems). Participants were not required to name their diagnosis correctly in order to score 2, but were required to provide some recognition that their memory problems were greater than the normal aging experience.

### Procedures

#### Coding procedure

The self-persistence interview, life story interview, and awareness of memory deficit interview were transcribed strict verbatim by a professional transcriber. They were ordered using blind, random numbering for the purposes of coding. A team of independent coders were involved in the coding the various elements of the present study: the self-persistence interview; the life stories for self-event connections, global coherence, and cultural life script. All coders were blind to group membership and, although they were aware of the general topic matter of the study, they were not aware of the hypotheses relating to the coding manuals they were applying. For each separate coding exercise, a single, primary coder was responsible for coding all of the transcripts. Coders were trained to the manuals by one of the authors (S.C.P.), and trial coding exercises were conducted using training examples to ensure that reliability was satisfactory before the transcripts were coded. For the awareness of memory deficits interview coding was completed by one of the authors (S.C.P.) a minimum of 6 months, and in most cases 2 years, following the interviews.

Reliability was established by using a second independent coder for each task, who double coded a random selection of 20% of the transcripts. For ordinal scales and those which generated a frequency count for each participant, the intraclass coefficient was used, while for nominal scales, Cohen's Kappa was used (Cohen, 1960; Landis and Koch, 1977). Reliability statistics for each coding element are set out in Table 4.

**Table 4:** Inter-rater reliability measures for double-coded transcripts.

	**α**		**α**
**Self-persistence interview**		**Life story interview**	
I-Self persistence	0.91	Temporal coherence	0.89
Me-self persistence	0.87	Causal coherence	0.88
Persistence explanation	0.96	Thematic coherence	0.75
		Self-event change connection	0.77
		Self-event stability connection	0.83
	α		**Kappa**
Awareness of memory deficits interview	1.0	Cultural life script event categories (present/absent)	Range:0.43–1.0

*α = Cronbach's alpha*.

#### General procedure

This study was part of a larger study examining the relationship between AM and sense of self, and thus were embedded in a more extensive procedure. The protocol was approved by the Northern Y Regional Ethics Committee, and carried out in accordance with the recommendations of the Ethical Guidelines for Observational Studies, National Advisory Committee on Health and Disability Support Services Ethics. All subjects gave written informed consent in accordance with the Declaration of Helsinki. Each participant was interviewed twice (each 1.5–2 h including breaks), with sessions held ~1 week apart.

Session 1 included introducing the study and gaining informed consent, a brief background interview, assessment of awareness of memory deficits, the MMSE, and the life story interview. Session 2 included verbal fluency (COWA), TST (present) and TST (Past), with the self-persistence interview administered after other intervening tasks.

### Statistical analyses

Statistical analyses were carried out using IBM SPSS Statistics 20 for Windows. Frequency data were analysed using Chi-square tests for independence. Analyses involving between and within-group differences were analysed using mixed-factorial Analyses of Variance (ANOVAs). When Mauchly's Test of Sphericity was violated, the Greenhouse-Geisser correction was used. Bonferroni pairwise comparisons were used to test for differences in significant within-subject main effects. For significant group effects, the Games and Howell (1976) *post-hoc* test procedure was used. As three groups were involved in these comparisons, significant interactions involving “group” were broken down using a two-step process. First, to determine which groups contributed to the interaction, either a series of Bonferroni-corrected one-way ANOVAs for each level of the other variable were run or mixed-factorial analyses were computed for each group pairing to determine which pairings contributed to the interaction (HC/aMCI, HC/AD, aMCI/AD). In each instance, the approach which resulted in the fewest comparisons that clarified the nature of the interaction was selected. Second, these omnibus ANOVAs were followed-up using Bonferroni or Games-Howell pairwise comparisons, as appropriate. For one-way ANOVAs where there was unequal variance between the groups, the Brown-Forsythe *F*-statistic was used (Field, 2009). Group differences in relation to ordinal data was assessed using Kruskal-Wallis non-parametric tests, followed up with pairwise comparisons using Mann-Whitney *U*-tests, correcting for multiple comparisons with a Bonferroni correction. Spearman's rho correlations were calculated for ordinal data. The significance of correlations were determined using a Holm-Bonferroni (sequentially-rejective) procedure (Holm, 1979) to correct for multiple comparisons.

## Results

### Diachronic unity: self persistence interview

One participant from each of the AD and aMCI groups was unable to complete the interview due to fatigue, leaving 25 HC, and 14 in each of the AD and aMCI groups.

#### Perceived persistence

Table 5 summarises responses to the question of whether they believed that they were still the same person as they were in their early 20s. A chi-square test (combining “yes and no” responses with “no” responses) revealed no significant relationship between group and type of response, χ^2^_(2)_ = 0.19, *p* = 0.91.

**Table 5:** Number (percentage) of participants responding “Yes” or “No” to the perceived persistence question of the self-persistence interview.

	**Yes**	**No**
HC group	11 (44%)	14 (56%)
aMCI group	7 (50%)	7 (50%)
AD group	7 (50%)	7 (50%)

*HC, Healthy control; aMCI, amnestic Mild Cognitive Impairment; AD, Alzheimer's disease*.

#### Persistence of the I-self and Me-self

**Figure 2:**
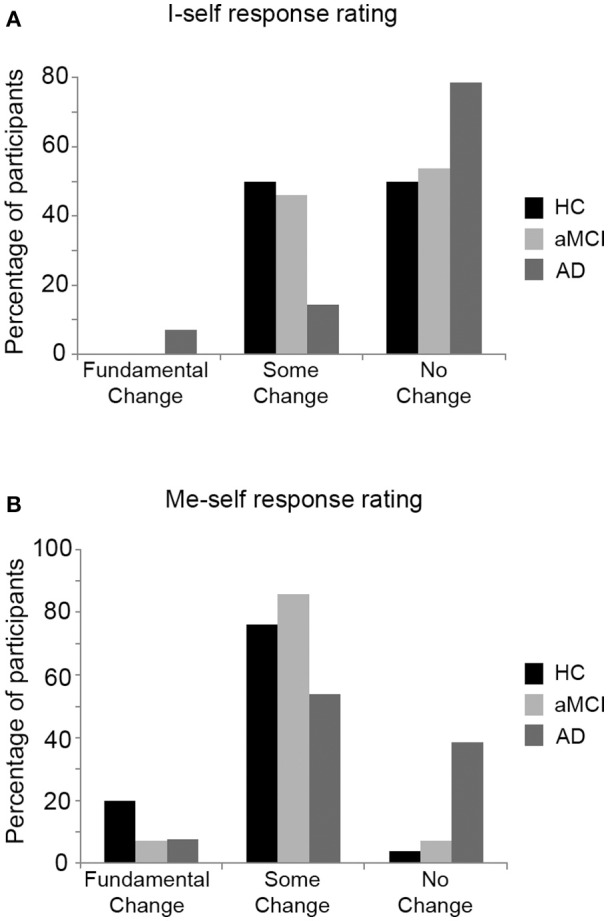
Percentage of participants from each group providing each response type on **(A)** the I-self rating scale, and **(B)** the Me-self rating scale of the self persistence interview. HC, Healthy control; aMCI, amnestic Mild Cognitive Impairment; AD, Alzheimer's disease.

Participant responses (when asked how they believed they had changed, and how they believed they had remained the same since they were in their early 20s) were separated into aspects which related to persistence of I-self (core) and me-self (traits and characteristics), and rated for whether these responses indicated no, some or fundamental change since their 20s. Figure 2 displays the distribution of each group in relation to these ratings. The responses of three participants could not be coded in relation to one of the subscales: two participants (one from each of the HC and aMCI groups) provided no information relevant to the I-self subscale, and one participant from the AD group provided no information relevant to the me-self subscale. These participants were excluded from the relevant portion of the analysis.

There was no significant difference between the groups in relation to the ratings for degree of I-self persistence expressed in their responses, *H*_(2)_ = 2.34, *p* = 0.31. There was, however, a significant difference between the groups in relation to the degree of me-self persistence expressed, *H*_(2)_ = 6.82, *p* = 0.03. Pairwise Mann-Whitney *U*-tests (Bonferroni-corrected alpha level set at *p* < 0.017) revealed that the AD group's responses tended to be rated as expressing greater me-self persistence than the responses of the HC group (*p* = 0.016). The degree of me-self persistence expressed in the responses of the aMCI group did not differ significantly from the HC or AD groups (all *p-*values ≤ 0.28).

#### Persistence explanation

**Figure 3:**
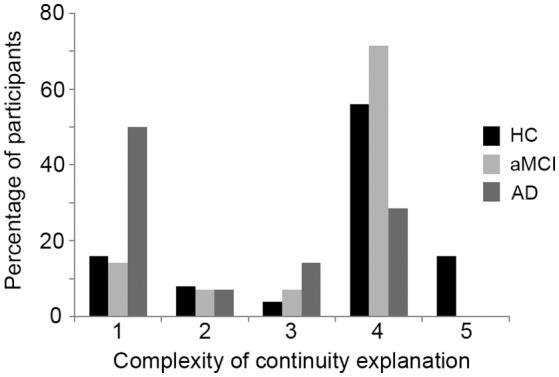
Percentage of participants from each group providing each level of sophistication of response on the persistence explanation. HC, Healthy control; aMCI, amnestic Mild Cognitive Impairment; AD, Alzheimer's disease. Scores of 1 represent the lowest level of sophistication, while scores of 5 indicate the highest level of sophistication.

The distribution of the level of sophistication of participants' persistence explanations for each group are shown in Figure 3. Of the AD group, 50% provided explanations for their persistence that were at the simplest level of sophistication, compared with 16% of the HC group and 14% of the aMCI group. Responses at this simplest level (level 1) focus on simple, surface attributes to account for self persistence while ignoring ways in which the individual may have changed, as illustrated by the following response from a participant in the AD group:

*If I was walking down the road, and saw someone bashing their dog or kid, I'd stop them. I've always done that. If someone was old and in a wheelchair, I'd help them. Always the same if someone needed help I'd help them*.

In contrast, around 70% of both the HC and aMCI groups provided responses at the highest two levels of sophistication, compared with just 30% of the AD group. Such responses either grappled with the realities of how the individual had changed while still identifying some core attributes which accounted for self-persistence (level 4), or weighed up many competing explanations for self-persistence (level 5), as illustrated by the following excerpt from a member of the aMCI group (scored level 4):

*My experience has increased but my physical self has decreased, aged. My brain problems/memory has changed my outlook. I get depressed sometimes. I am not enjoying looking forward. [My parents]… set standards for me. Not deeply religious, but stronger religious feelings than I had and they brought me up on those standards. And that has been a strong steer for me through my life. Attitudes and values*.

The groups differed significantly in the sophistication of their persistence explanations, *H*_(2)_ = 8.8, *p* = 0.01, with pairwise Mann-Whitney *U*-tests (Bonferroni-corrected alpha level set at *p* < 0.017) confirming that the AD group's persistence explanations were significantly less sophisticated (*Mdn* = 1.5; mean rank = 17.54) than those of the HC group (*Mdn* = 4; mean rank = 31.26; *p* = 0.01). There was also a non-significant trend for the AD group to have lower scores than the aMCI group (*Mdn* = 4*;* mean rank = 28.86; *p* = 0.02). No significant difference was found in the scores for the aMCI and HC groups (*p* = 0.57*)*.

A possible criticism of this method of assessing diachronic unity is that the task is a very difficult one, and the lower sophistication in the AD group's persistence explanations could relate to more general disease-related deterioration including general cognitive decline, loss of verbal fluency, or lack of insight. However, no significant Spearman's correlations were found between the sophistication of persistence explanation scores and MMSE, COWA, or awareness of memory deficits in the AD group (all *p-*values > 0.12).

Taken together, these analyses indicate that while the AD group demonstrated high confidence in their beliefs about their persistence over time (perceived persistence, I-self persistence, and me-self persistence), their ability to construct a sophisticated justification for their persistence across time was impaired. In fact, Spearman's rho correlations within the AD group revealed a significant negative correlation between the persistence explanation and perceived persistence, *r*_*s*_ = −0.61, *p* = 0.019, as well as a marginally-significant negative correlation between the persistence explanation and I-self persistence, *r*_*s*_ = −0.54, *p* = 0.047. In other words, those within the AD group who provided more sophisticated explanations for their persistence also tended to express *less* certainty over whether they remained the same person over time. This observation suggests that perceived and I-self (core) persistence may represent different facets of diachronic unity from the persistence explanation.

### Semantic continuity: life story interview

#### Self-event connections

**Figure 4:**
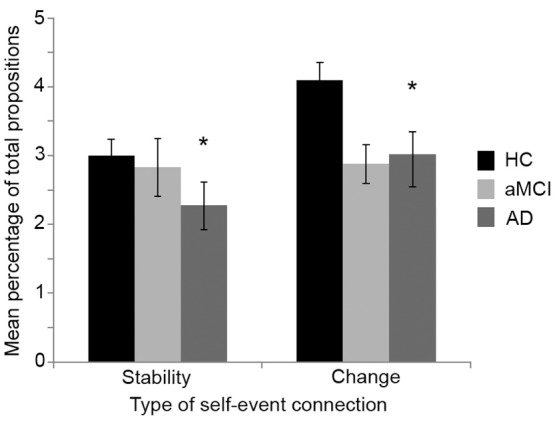
Mean number of self-event connections in the life stories as a percentage of the total number of propositions for each group. HC, Healthy control; aMCI, amnestic Mild Cognitive Impairment; AD, Alzheimer's disease. Error bars denote one standard error of the mean. ^*^AD group significantly lower than HC group.

All participants completed the life story interview. To compare the amount and type of autobiographical reasoning used in the life stories, a mixed-factorial ANOVA was conducted with life period (childhood, early adulthood, middle adulthood, late adulthood) and type of self-event connection (change, stability) as the within-subjects factors, and group (HC, aMCI, AD) as the between-subjects factor. The dependent variable was the number of self-event connections in each chapter as a percentage of the total number of propositions used in that chapter (see Figure 4).

There was a significant main effect for group, *F*_(2, 52)_ = 4.7, *p* = 0.01, with *post-hoc* Games-Howell comparisons showing that the AD group used a significantly lower percentage of self-event connections in their life stories (*M* = 5.31, *SE* = 0.52) compared with the HC group (*M* = 7.10, SE = 0.36; *p* = 0.02). The aMCI group did not differ significantly from either the HC or AD groups (*M* = 5.70, *SE* = 0.56; both *p*-values ≥ 0.16). There was also a significant main effect for type of self-event connection, *F*_(1, 52)_ = 5.74, *p* = 0.02, a larger percentage of change connections (*M* = 3.47, *SE* = 0.19) used than stability connections (*M* = 2.76, *SE* = 0.18). There were no other significant main effects or interactions (all *p-*values ≥ 0.12).

#### Global coherence

A mixed ANOVA examined the difference between the groups on the global coherence of their stories, with type of coherence (temporal, causal, thematic) and life period (childhood, early, middle, and late adulthood) as the within-subject factors and group (HC, aMCI, AD) as the between-subject factor. There was a significant main effect for group, *F*_(2, 52)_ = 19.64, *p* < 0.001, with the stories of the AD group rated significantly less coherent than either the HC or aMCI groups (Games-Howell *post-hoc* tests: all *p-*values < 0.001) while the aMCI and HC groups did not differ (*p* = 0.44). There was also a significant main effect for life period, *F*_(3, 156)_ = 1.11, *p* < 0.001, with the early adulthood period showing greater coherence than other chapters (all *p-*values < 0.01).

**Figure 5:**
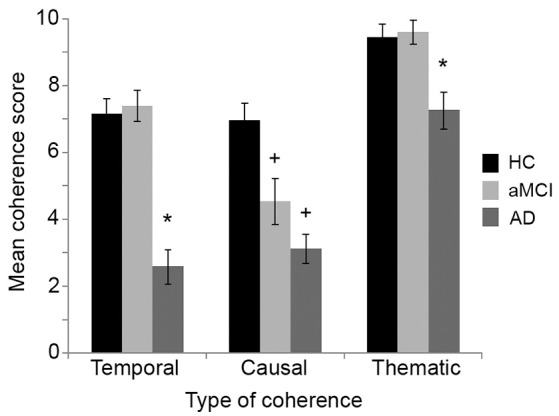
Mean score for each type of coherence (temporal, causal, and thematic) across the whole life story, for each group. HC, Healthy control; aMCI, amnestic Mild Cognitive Impairment; AD, Alzheimer's disease. Errors bars denote one standard error around the mean. ^*^AD significantly lower than HC/aMCI groups; +Significantly lower than HC group.

The main effect for type of coherence was also significant, *F*_(2, 104)_ = 101.85, *p* < 0.001, but was modified by a significant interaction between group and type of coherence, *F*_(4, 104)_ = 9.00, *p* < 0.001 (see Figure 5). The analysis was rerun for each group pairing, and the interaction remained significant for all three pairs (all *p-*values ≤ 0.001). Pairwise comparisons showed that the HC and aMCI groups scored higher than the AD group on both temporal and thematic coherence (all *p-*values ≤ 0.004). In relation to causal coherence, however, both the AD and aMCI groups scored lower than the HC group (both *p-*values ≤ 0.01). All three groups scored significantly higher on thematic compared with causal or temporal coherence (all *p-*values < 0.001), but only the aMCI group scored significantly lower on causal than temporal coherence (*p-*value < 0.001). No other pairwise comparisons were significant (*p* = 1.00). These results indicate that while the AD group scored lower on all forms of coherence, the aMCI group showed a specific deficit in relation to causal coherence.

#### Cultural life script

Separate one-way ANOVAs conducted on the number of cultural life event categories sampled and the total number of cultural life events mentioned in the life story. At a Bonferroni-corrected alpha level (0.025) there was no significant group difference in the number of cultural life script categories sampled, *F*_(2, 32.39)_ = 1.89, *p* = 0.17, or the number of events used in the life stories, *F*_(2, 52)_ = 3.41, *p* = 0.04, although the latter approached significance. When the analyses were rerun using the number of cultural life script events and event categories sampled as a percentage of the total number of propositions used in the life stories, there was no significant difference between the groups on either number of categories sampled, *F*_(2, 25.63)_ = 1.38, *p* = 0.27, or the total number of events used in the life stories, *F*_(2, 27.86)_ = 0.80, *p* = 0.46 (Brown-Forsythe). These findings indicate that the memory-impaired groups did not differ from healthy controls in their use of cultural life script events in their stories.

### Diachronic unity and semantic continuity

Next we examined the relationship between measures of diachronic unity and semantic continuity in the AD group as these participants had the most severe memory impairments. Spearman's rho correlations were conducted between three measures from the self-persistence interview (I-self persistence, me-self persistence, persistence explanation) and five measures from the life story interview (the number of self-event stability and change connections used in the life story, and ratings for temporal, causal, and thematic coherence). To control for differences in the length of the life stories, self-event connection measures were percentages of the total number of propositions used in the life story. Five of the 15 correlations were, or approached, significance (Holm-Bonferroni correction, first-level adjusted alpha, *p* < 0.003; second-level adjusted alpha, *p* < 0.004). Two of these were related to perceived persistence: a significant negative correlation with causal coherence, *r*_*s*_ = −0.84, *p* < 0.001, and a marginally-significant negative correlation with the number of self-event stability connections, *r*_*s*_ = −0.62, *p* = 0.02. In other words, AD participants who produced stories that were more causally coherent and contained higher percentages of self-event connections were also more inclined to state that they were not the same person they were in their early 20s. There was also a consistent pattern of marginally significant positive correlations between the persistence explanation and quality of narrative identity. More sophisticated explanations were associated with the greater use of self-event stability connections, *r*_*s*_ = 0.72, *p* = 0.004, and greater temporal, *r*_*s*_ = 0.58, *p* = 0.03, and causal coherence, *r*_*s*_ = 0.54, *p* = 0.049. This indicates that AD participants with better narrative identity also tended to provide more complex explanations for self-persistence. When the same correlations were conducted within the HC group, as expected there were no significant associations.

## Discussion

This study provides one of the first systematic investigations of diachronic unity in memory-impaired older adults. In line with our model of how AM supports diachronic unity (Prebble et al., 2013; Figure 1), it is possible that for individuals with disorders characterised by impaired autonoetic consciousness (Rauchs et al., 2007; Ally et al., 2009; Hudon et al., 2009; Irish et al., 2010), subjective beliefs about their persistence across time (diachronic unity) are maintained, and supported by semantic continuity. We investigated this hypothesis in aMCI and AD by assessing the integrity of, and relationship between, diachronic unity and semantic continuity (narrative processes such as autobiographical reasoning and global coherence including personal temporal chronology).

### Diachronic unity in aMCI and AD

A central finding of this study was that the memory-impaired groups did not differ significantly from healthy older participants in relation to their subjective beliefs about their diachronic unity (i.e., perceived persistence and I-self persistence). About half of healthy and memory-impaired participants expressed strong views that they remained the same person as they were in their early 20s. This finding was accompanied by a preserved sense of persistence of their core essence, which is consistent with qualitative evidence from previous studies that those with memory impairments may nevertheless retain a deep conviction about their persistence across time (Medved and Brockmeier, 2008; Rathbone et al., 2009; Philippi et al., 2012).

Individuals with aMCI provided explanations for their preserved beliefs of persistence that were as sophisticated as those generated by healthy controls, as measured using Chandler et al.'s (2003) continuity explanation. In contrast, in AD these preserved beliefs about diachronic unity were explained in a significantly less sophisticated way than both the HC and aMCI groups. Specifically, half of the AD group provided answers that relied on superficial, surface characteristics to justify stability, while ignoring or downplaying aspects of personal change. Importantly, this difference was not simply related to global cognitive decline or reduced fluency. In contrast to Chandler et al.'s findings with suicidal youth, our results suggest that a coherent and sophisticated rationale for one's persistence is not necessary to maintain a strong inner conviction that you continue to be the same person across time; in memory-impaired older adults even very simple persistence explanations may be sufficient to sustain beliefs about one's diachronic unity.

In fact, for those in the AD group, less sophisticated persistence explanations were associated with *greater* certainty about their diachronic unity (subjective persistence over time, including their core I-self). One possible explanation is that simplistic, “change ignoring” persistence explanations provide a way to support a sense of diachronic unity without needing to grapple with the realities of personal change, focusing instead on superficial aspects of personal consistency, as illustrated by the following response:

*I can't see myself being very different now. I just am like I am. If people talk to me I'll talk to them. I'm a mother, I help people.…I just seem to do the same things. If people talk to me I talk to them, if people need help I help them. Always been the same*.

Focusing on superficial aspects of consistency may be a useful strategy for maintaining a strong conviction about core persistence in the face of memory loss. The AD group were significantly more likely to emphasise greater persistence in the me-self over time (i.e., traits and characteristics) than healthy controls. A related suggestion has been made by Clare et al. (Clare, 2003, 2004; Naylor and Clare, 2008) in relation to lack of awareness about memory deficits. They suggest that reduced awareness may provide an important strategy for reducing threats to identity for certain individuals. In this study, however, there was no significant correlation between our coarse measure of lack of awareness of memory deficits and the use of more simplistic persistence explanations. It is also possible that the focus on stability rather than change in the responses of the AD group may relate to their memory deficits, and an inability to recollect the nuanced ways they have changed and stayed the same across the lifespan.

Another possible account of these findings is that the perceived persistence and I-self persistence measures used in the present study do not directly assess beliefs about core diachronic unity, but rather assess the level of engagement that a participant has with difficult questions about self-persistence. The direct questions regarding persistence used in the present study (e.g., do you believe you are the same person? In what ways have you changed?) appear to directly address questions about self-persistence, yet someone could respond to these questions in a manner that indicates essential changes to who they are while nevertheless continuing to believe that these changes have occurred to the same, unifying “me.” When individuals express less conviction about their core persistence on these measures, for example, by describing themselves as being “a fundamentally different person,” this may not be a marker for uncertainty or degradation in their beliefs about core persistence, but rather an indication of intelligent engagement with the enigmatic challenges of diachronic unity. It is difficult to know how to distinguish such an answer from one in which the individual has lost the ability to see themselves as the same unified entity extending across time—a true loss of diachronic unity. In this study those individuals with memory-impairment who indicated doubts about their diachronic unity (thus scoring lower on perceived and I-self persistence), tended to produce more sophisticated explanations for their diachronic unity, which indicates they did engage with the questions about self-persistence. We suggest they may have been attempting to explore the difficult questions of change and persistence over the lifetime at a deep, level, which in turn allowed them to produce sophisticated explanations for the changes they perceived in their fundamental diachronic unity.

These issues indicate that further methodological work is needed to determine how best to assess diachronic unity. In particular, research is needed with groups who are likely to have true disruptions in their subjective beliefs about self-persistence (e.g., suicidal populations, Chandler et al.'s, 2003; Hsiao et al., 2013) in order to determine whether they provide qualitatively different types of answers regarding their diachronic unity.

### Semantic continuity in aMCI and AD

This study revealed that while many aspects of semantic continuity were preserved in aMCI, there were a number of differences in the semantic continuity of individuals with AD and healthy older adults. First, the life stories of the aMCI group were similar to those of healthy controls in terms of inclusion of cultural life script events, as well as levels of temporal and thematic coherence. In particular, the preservation of temporal coherence indicated that the ability to construct personal temporal chronology is relatively preserved in aMCI. Moreover, aMCI individuals appeared to engage in autobiographical reasoning, although the number of self-event connections they generated was at a level intermediate between that of the healthy control and AD groups (differing significantly from neither group).

Interestingly, however, there was a significant reduction in the causal coherence of the life stories generated by the aMCI group, indicating that they were less able to explain the causal links between events, or between these events and their self-concept. This finding may indicate that causal coherence, at least as it was measured in the present investigation, requires a more complex form of reasoning than the other types of coherence, and is affected earlier in the disease process. Habermas and de Silveira (2008) report that causal coherence develops steeply during adolescence, and is more complex than temporal coherence. Although they suggest that causal coherence is less complex than thematic coherence, and demonstrate that thematic coherence does not steeply increase until after age 16, it is interesting to note that their findings suggest causal coherence is almost absent in the stories of younger children, while a basic level of thematic coherence is present.

Alternatively, it is possible that the impaired causal coherence of the aMCI group may relate to the acute awareness that many possessed about their memory loss, leading to a difference in the way these individuals approached their stories. For example, in attempting to remember all of the details of their stories, they may have focused more deliberately on including important dates and events than on providing any meta-narrative to link the stories together. In addition, the effort of producing these life stories, including trying to remember the events and their temporal order, may have required greater cognitive energy for those with aMCI, leaving less to expend on more complex aspects of the story like causally linking events. Future work could explore the underlying basis of the reduced causal coherence in aMCI groups by using a guided autobiographical reasoning assessment with prompts to elicit these different reasoning processes.

In contrast to the limited change in semantic continuity in the aMCI group, there was a more general reduction in the quality of the narratives produced by the AD group. This included less evidence of autobiographical reasoning, with inclusion of fewer (as well as a lower percentage of) self-event connections about change and stability in their life stories. Moreover, there were reductions in temporal, causal and thematic coherence. These findings indicate that the AD group were making fewer links between the events they included in their stories and their self-concept, and that they were less able to convey the temporal order of events, the causal links between the elements of the story, and the overarching motifs and lessons that wove the story together. It may be that just as episodic memories deteriorate in AD, so too does the ability to link and integrate elements within the story into a coherent narrative.

The present findings also revealed some deterioration in the personal temporal chronology of those with AD. Consistent with reports that the narratives of those with AD may lack chronological ordering (Usita et al., 1998), the life stories of the present AD group were rated as having lower temporal coherence than the stories of the other groups. This was evident despite the provision of a broad temporal structure by dividing the life story into four sequential chapters, indicating deterioration in the ability to convey the absolute and relative temporal ordering of events through their life narratives. While this could relate to difficulties in ordering and sequencing events in the process of story construction, an interesting possibility is that this difficulty reflects a disruption in the temporal organisation of AM itself.

Despite this, the use of the cultural life script was well-preserved in the life stories of those with AD. Although there was a non-significant trend for those with AD to use fewer cultural life script events, after taking into account the length of their stories, there was no difference between the groups in relation to the number of cultural life script events, or event categories, used. This finding differs from that of Usita et al. (1998), but is consistent with those of Fromholt and Larsen (1991) and suggests that in AD cultural knowledge about the events to include in a life story remains largely intact.

These findings suggest that the organisational structure provided by the cultural life script, and the memories linked to it, may be relatively more resilient than other types of AM, perhaps due to the important socio-cultural function of these memories. This culturally-generated script may play an important role in structuring and organising the last remnants of AM in AD (Berntsen and Rubin, 2004). It is important to note, however, that the measure used in the present study only addressed whether the events were mentioned in the story, and not the detail or accuracy of the stories. It is possible that AD participants retained knowledge about the script (i.e., awareness that a given type of event should be included in their life story) but not any accuracy or detail about the event itself within their own life. The resilience of cultural life script events may also be due to the list of events being weighted to the first half of life, and particularly the early-adulthood “reminiscence bump” period (Berntsen and Rubin, 2004). To assess these possibilities, future investigations should explore the detail and accuracy of the cultural life events included in the stories of those with AD, and whether there is any difference in the recall of cultural life events from different life periods.

### Relationship between semantic continuity and diachronic unity

The present findings suggest a complex relationship between semantic continuity and diachronic unity. There was a consistent pattern of positive, marginally significant correlations between the sophistication of the persistence explanation and the quality of semantic continuity within the AD group, including the greater use of self-event stability connections, and greater temporal and causal coherence of life stories. This finding provides tentative support for the view that the ability to construct a quality life story, which links one's AMs into a coherent narrative that explains the development of the self across time, is associated with more sophisticated explanations for why one continues to remain the same person. Additional evidence for this notion comes from the aMCI group who had preserved autobiographical reasoning and temporal/thematic coherence of their life stories, and were able to generate persistence explanations as sophisticated as those produced by health controls. When considered together with the disruptions to phenomenological re-experiencing evident in aMCI (Ally et al., 2009; Hudon et al., 2009; Irish et al., 2010), this pattern of findings provides support for our model's prediction that intact semantic continuity is sufficient to support diachronic unity (Prebble et al., 2013).

An intriguing possibility, in line with the present model, is that the association of semantic continuity and diachronic unity reflects a causal relationship: that the ability to weave a high-quality life narrative scaffolds the ability to understand and explain one's persistence across time. The fact that this relationship was present in the AD group, but not the healthy controls is consistent with Habermas and Köber's (2014, 2015) finding and suggestion that autobiographical reasoning may be necessary for reflecting upon, and understanding, diachronic unity only when there are disruptions to self-persistence. A diagnosis of AD, and the accompanying personal and circumstantial changes, may constitute such a disruption. In the face of such disruptions, any deterioration in the ability to weave AM into a coherent narrative may contribute to an impaired ability to understand, at a higher conceptual level, why they remain continuous beings over time.

It is also possible, however, that what may underlie these relationships is cognitive decline in AD which is not directly related to memory, but affects both the construction of quality life narratives and successful engagement in the self-persistence explanation task. However, the loss of sophistication in self-persistence explanation in the AD group was not significantly correlated with general cognitive decline (MMSE) or verbal fluency (COWA), suggesting that other factors were involved. Nevertheless, other more subtle reasoning processes, not captured by simple measures like the MMSE, may be affected. To assess this possibility, future studies could use a control paradigm that requires a similar, but non-self-related reasoning exercise (e.g., explaining the persistence of animals or inanimate objects over time) in order to determine whether it is the ability to engage in such logical reasoning processes that is affected, or whether there are specific deficits in explaining one's own persistence.

Although better semantic continuity was associated with increased sophistication of self-persistence explanation, it was not associated with greater certainty about diachronic unity. On the contrary, in the AD group a significant negative relationship was found between the causal coherence of life stories and certainty about whether they remained the same person. This finding parallels those reported above in relation to phenomenological continuity: better memory performance (in this case, better integration of AM into a life narrative) was associated with more sophisticated explanations about, but less certainty of, self-persistence.

A limitation of the current investigation is its focus on past-facing diachronic unity and continuity. Recent theories have postulated that the adaptive function of the episodic memory system may not relate to reliving the past, but rather in being able to imagine, and therefore plan for, the future (Tulving, 2005; Suddendorf et al., 2009; Tulving and Szpunar, 2009), and neuroscientific work has established a connection between the ability to remember one's past and the projection of oneself forward into the imagined future (Schacter and Addis, 2007; Addis et al., 2008). It may be that the ability to mentally project oneself forward in time, and/or construct a story about the future self, plays a vital role in phenomenological and semantic forms of continuity and thus also diachronic unity.

While research into future thinking has focused primarily on the role of episodic memory, there are studies suggesting that semanticised AM may play an equally important role. Patients with semantic dementia, who have profound semantic memory deficits but largely preserved episodic memory, have been found to be as deficient at imagining personalised future scenarios as individuals with AD, suggesting that semanticised AM may provide the scaffolding that allows episodic memory details to be recombined into novel future scenarios (Irish et al., 2012). There is also evidence that fundamental breakdowns in the sense of semantic continuity in semantic dementia can arise from problems with the ability to mentally project oneself forward in time, and may be associated with suicidal behaviour (Hsiao et al., 2013). This future-facing aspect of continuity and diachronic unity, and its association with semantic and episodic memory ability, is an important area for future investigation.

## Summary

This study provides some important insights for our model of how AM supports diachronic unity by examining whether semantic continuity can provide a sense of self persistence in disorders where AM deficits disrupt phenomenological continuity (Prebble et al., 2013). The findings in both aMCI and AD support the predicted importance of semantic continuity to diachronic unity. In aMCI, the quality of life narratives was largely preserved, as were measures of self-persistence, indicating that even when the continuity afforded by phenomenological re-experiencing is disrupted, semantic continuity is sufficient to support diachronic unity. However, the findings from the AD group suggest a more nuanced relationship than was originally proposed in our model. Underlying the complex pattern of findings was a divergence between the two aspects of diachronic unity in the AD group: beliefs about the persistence of the core self over time (i.e., perceived and I-self persistence) vs. the sophistication of persistence explanations. The AD group had maintained the former, but showed deterioration in the latter. Within the AD group, those who scored higher on measures of persistence of the core self also provided less sophisticated explanations for their self-persistence and were less able to use their AM as a vehicle to explore how they came to be the person they are through their life narratives (i.e., poorer semantic continuity).

These apparently paradoxical findings indicate a multifaceted relation between diachronic unity and semantic continuity in individuals lacking phenomenological continuity as occurs in AD. Those individuals with AD who retain better quality semantic continuity and life narratives, including better autobiographical reasoning and greater global coherence, are able to produce a more complex persistence explanation. This is accompanied by less conviction about the persistence of the core self, without incorporating some degree of fundamental change. Thus, better semantic continuity in individuals with AD may facilitate deep engagement with issues of self-persistence, and this may be reflected both by more sophisticated responses on the persistence explanation, and by responses to questions about diachronic unity that indicate an appreciation of personal change across the lifespan.

A poor quality life narrative that nevertheless contains regular features of a cultural life script, appears to be sufficient for individuals with AD to perceive familiarity with their past and thus maintain strong subjective beliefs of self-persistence over time. Nevertheless, when the connection with one's remembered past is diminished by the combination of poor phenomenological and semantic continuity, this may contribute to a less sophisticated understanding about personal persistence; one which over-emphasises superficial aspects of stability and fails to integrate an understanding of change. Further work is needed to investigate whether this apparent lack of engagement with the paradox of diachronic unity in individuals with AD and diminished semantic continuity is due to an inability to remember personal change, or may serve as an important protective mechanism for the self-persistence of those with AD in the face of tremendous personal change.

## Author contributions

LT, SP, and DA conceived of and designed the study. SP collected and analysed the data under the supervision of LT and DA while she was undertaking her PhD. LT and SP drafted the manuscript with input from DA.

### Conflict of interest statement

The authors declare that the research was conducted in the absence of any commercial or financial relationships that could be construed as a potential conflict of interest.

## Footnotes

^1^Originally called “perceived continuity” (Troll and Skaff, 1997).

^2^Originally called the “continuity of the I-self and Me-self” (Troll and Skaff, 1997).

^3^Originally called the “continuity explanation” measure (Chandler et al.'s, 2003).

## Appendix A

### Glossary

*Autobiographical memory:* Memory that is personal in nature in that it involves the self (e.g., personally-experienced events, facts about the self).

*Autobiographical reasoning:* A narrative process that involves creating connections between different parts of the life story (e.g., events in the past, present, and future) and the self and can emphasise stability or change; this process serves to increase the temporal, causal, and thematic coherence of the life story.

*Autonoetic consciousness:* The sense of consciously re-experiencing a past event; a critical feature of episodic memory.

*Diachronic unity:* The unification of the self as a single entity that persists across time; self-persistence.

*Episodic memory:* A form of autobiographical memory that represents specific events that one experienced in the past.

*I-self:* the essential, core self, the underlying essence of who you are, the inner entity; related to phenomenological continuity.

*Me-self:* the attributes one uses to describe oneself, e.g., traits, attributes, physical, and personality descriptions, behaviour, likes and dislikes, values and beliefs; related to beliefs about semantic continuity. *Narrative:* Coherent and meaningful life story that convey “how I was and how I came to be who I am” that highlights the temporally-extended self-concept and personal temporal chronology.

*Narrative identity:* Sense of identity emergent from the process of constructing a life story.

*Phenomenological continuity:* The subjective experience of the self-existing over time, arising from episodic remembering and autonoetic consciousness.

*Self-persistence:* See diachronic unity.

*Semantic continuity:* The knowledge that the self exists over time; a by-product of the construction of a coherent, temporally-extended narrative.

*Semantic memory:* An abstract, conceptual form of autobiographical memory that synthesises large amounts of knowledge and facts about the self, including self-concept (e.g., traits, preferences) and knowledge of past experiences, etc.

*Temporally-extended self-concept:* The self-concept (e.g., trait-self knowledge, preferences, behaviours) in the past, present and future; incorporates change and stability of self-concept.

*Personal temporal chronology:* The temporal sequence of events experienced in one's life; can be measured by temporal coherence of life narratives.
